# Rheological Properties of Different Graphene Nanomaterials in Biological Media

**DOI:** 10.3390/ma15103593

**Published:** 2022-05-18

**Authors:** Arisbel Cerpa-Naranjo, Javier Pérez-Piñeiro, Pablo Navajas-Chocarro, Mariana P. Arce, Isabel Lado-Touriño, Niurka Barrios-Bermúdez, Rodrigo Moreno, María Luisa Rojas-Cervantes

**Affiliations:** 1 School of Architecture, Engineering and Design, European University of Madrid, C: Tajo s/n. Villaviciosa de Odón, 28670 Madrid, Spain; chokinavajas@gmail.com (P.N.-C.); mariana.arce@universidadeuropea.es (M.P.A.); misabel.lado@universidadeuropea.es (I.L.-T.); niurka.barrios@universidadeuropea.es (N.B.-B.); 2 Institute of Ceramics and Glass (ICV-CSIC), 28049 Madrid, Spain; rmoreno@icv.csic.es; 3 Department of Inorganic and Technical Chemistry, Universidad Nacional de Educación a Distancia (UNED), Urbanización Monterrozas, Las Rozas, 28232 Madrid, Spain; mrojas@ccia.uned.es

**Keywords:** rheology, carbon-based nanomaterials, biological fluids, fetal bovine serum, bovine serum albumin

## Abstract

Carbon nanomaterials have received increased attention in the last few years due to their potential applications in several areas. In medicine, for example, these nanomaterials could be used as contrast agents, drug transporters, and tissue regenerators or in gene therapy. This makes it necessary to know the behavior of carbon nanomaterials in biological media to assure good fluidity and the absence of deleterious effects on human health. In this work, the rheological characterization of different graphene nanomaterials in fetal bovine serum and other fluids, such as bovine serum albumin and water, is studied using rotational and microfluidic chip rheometry. Graphene oxide, graphene nanoplatelets, and expanded graphene oxide at concentrations between 1 and 3 mg/mL and temperatures in the 25–40 °C range were used. The suspensions were also characterized by transmission and scanning electron microscopy and atomic force microscopy, and the results show a high tendency to aggregation and reveals that there is a protein–nanomaterial interaction. Although rotational rheometry is customarily used, it cannot provide reliable measurements in low viscosity samples, showing an apparent shear thickening, whereas capillary viscometers need transparent samples; therefore, microfluidic technology appears to be a suitable method to measure low viscosity, non-transparent Newtonian fluids, as it is able to determine small variations in viscosity. No significant changes in viscosity are found within the solid concentration range studied but it decreases between 1.1 and 0.6 mPa·s when the temperature raises from 25 to 40 °C.

## 1. Introduction

Graphene, which was first prepared in 2004 by K.S. Novoselov et al., is a two-dimensional carbon sheet comprised of sp^2^ hybridized carbon atoms packed in a regular atomic-scale hexagonal structure [1]. This material has attracted great interest due to its extraordinary mechanical properties, such as high fracture strength and Young’s modulus, excellent electrical and thermal conductivity, quick charge carrier mobility, large specific surface area, and good biocompatibility [2].

Graphene-based materials, such as graphene oxide (GO) or graphene nanoplatelets (GNP), maintain many of the magnificent and interesting properties of graphene. The oxidized form of graphene, GO, contains several reactive oxygen functional groups on the surface that enhance its dispersion and stability in aqueous and organic solvents [3]. GO is prepared via a simple and low-cost route using graphite, an abundant and inexpensive natural resource [4], and it is highly favorable for bio applications due to its large surface, good dispersibility in water, easy surface functionalization and good biocompatibility [5,6]. Graphene nanoplatelets are two-dimensional structures with an average thickness of 5 to 10 nanometers and a specific surface area of 50 to 750 m^2^/g, which have great interest due to their excellent electrical conductivity and high mechanical properties [7,8].

Graphenes are used in a broad variety of applications, including quantum physics, nanoelectronics, catalysis, nanocomposites, and sensor technology [9,10]. It has been also reported that they exhibit strong antibacterial toxicity [11,12] and are very useful in several medical applications, including therapeutics, diagnostics, and regenerative medicine [13,14]; therefore, one main focal point of the application of graphene-based materials is in the field of nanomedicine. Many studies have attempted to exploit them for drug delivery or imaging, since they can solubilize and bind drug molecules because of their large surface area and delocalized π electrons [13,15]. However, with the purpose of using graphene derivatives in the field of biomedicine, it is important to know the behavior of these materials in biological media. In this sense, the oxygen functional groups of graphene oxide provide attachment sites to various biological molecules [16]. Upon contact with the biological environment, the nanomaterial is immediately coated by a layer of proteins that form the protein-corona. The structure of this complex is of vital importance for understanding the interaction of such nanomaterials with biological systems [17,18]. Regarding the nanomaterial, the surface change, adsorption potential, particle size and shape are among the main properties that can influence the specific binding of proteins [19]. It is also necessary to consider that proteins adsorbed on the nanomaterial surface can lose their native conformation, and consequently, their function [20].

The physical adsorption of proteins onto the surface of graphene oxide is achieved through non-covalent interactions. The interactions involved in protein–GO/rGO complex formation are electrostatic interactions, hydrogen bonding, hydrophobic interactions, van der Waals forces, and π–π stacking interactions. The non-specific interactions are mainly controlled by the layer content and interfacial stress transfer between the graphene oxide layers and the protein structure. 

Bovine serum albumin (BSA) is commonly used as a model protein due to its high percentage of sequence identities compared to human serum albumin (HSA, 76%), low cost and high structural stability [21]. BSA is a kind of globular protein consisting of a single peptide chain with 583 amino acids. It can be loaded on a variety of substrates, which contain cations, anions, amino-acids and so on. The adsorption process of BSA onto the GO layers is mainly driven by the mutual electrostatic interaction between the negatively charged GO sheets and positively charged -NH_2_ group on BSA chains, together with some hydrogen bonding [22]. The BSA conformation and adsorption behavior on the GO surface under various pH conditions was studied by Wu and coworkers [23], who showed that the adsorption mechanism is mainly controlled by the protein conformational change and electrostatic and hydrophobic interactions, and clear secondary structural perturbations were observed upon interaction with the GO surface. This physiological protein is frequently employed as an effective vehicle in tissue engineering and drug delivery systems due to its biodegradability, non-antigenicity, non-toxicity, and easy production [24]. Fetal bovine serum (FBS), whose main constituent is BSA, is the most widely used growth supplement for cell cultures, primarily because of its high levels of growth stimulatory factors and low levels growth inhibitory factors [25].

Some works have evaluated the rheological properties of the protein solutions [26,27,28], but the main issue is to maintain the same viscosities of the physiological medium when the graphene-based nanoparticles are added, since changes in the viscosity of the protein solutions can affect to their structure. There are some works reporting the rheological behavior of nanoparticles in different media [29,30], in which the effect of various factors, such as preparation conditions, nanoparticle and base fluid properties, concentration, temperature, surface charge, and aggregation state on the rheological behavior of nanofluids is discussed. In previous studies, Cerpa et al. checked the behavior of single-walled carbon nanotubes (SWCNTs) and multi-walled carbon nanotubes (MWCNTs) in contact with biological fluids, focusing the interaction between their surface and the protein chains [31,32]. The suspensions showed Newtonian flow behavior and the viscosity values of SWCNT suspensions in water and FBS at 1.5 mg/mL were less than 3 mPa·s. In addition, the viscosity range is wider for the different MWCNTs studied (2 and 6 mPa·s) compared with the results obtained for SWCNTs, whose viscosity values are lower (<3 mPa·s) for a shear rate of 1000 s^−1^. In contrast, when the dispersion medium is FBS, the viscosity range of MWCNTs is narrower, between 1.8 and 2.8 mPa·s. The same happens when comparing with the values obtained for the SWCNTs. However, to the best of our knowledge, no studies on the rheological behavior of graphene-related materials in biological media have been reported in the literature. Considering all the above, in this work, a structural and rheological characterization of the interaction of three nanomaterials based on graphene, i.e., graphene oxide (GO), graphene nanoplatelets (GNP) and expanded graphene oxide (EGO) with two different biological media, BSA and FBS, has been performed. 

## 2. Materials and Methods

### 2.1. Materials

Graphene oxide (GO) and graphene nanoplatelets (GNP) (NIT GRAPHENIT-OX) were obtained from Nanoinnova Technologies S.L, Illescas, Spain. GO has a specific surface area of 20–35 m^2^/g and median mesoporous pore diameter of 12.8 nm. GNPs have a degree of oxidation of 2%, a surface area of 101 m^2^/g, a density of 2 g/cm^3^ and lateral dimension of about 2–3 micron with less than 5 layers thick. Expanded graphene oxide (EGO) was obtained from the Chemical Engineering Laboratory of the University of Castilla La Mancha, Ciudad Real, Spain. It was synthesized following the procedure described elsewhere [33] based on the pilot method reported by Lee et al. [34]. EGO has a specific surface area of 116 m^2^/g and median mesoporous pore diameter of 22 nm. Biological fluids, bovine fetal serum (FBS, South America) and bovine serum albumin (BSA, USA) were obtained from Gibco (Thermo Scientific, Paisley, UK) and Sigma Aldrich, Saint Louis, MO, USA, respectively.

### 2.2. Dispersions Preparation

**Figure 1:**
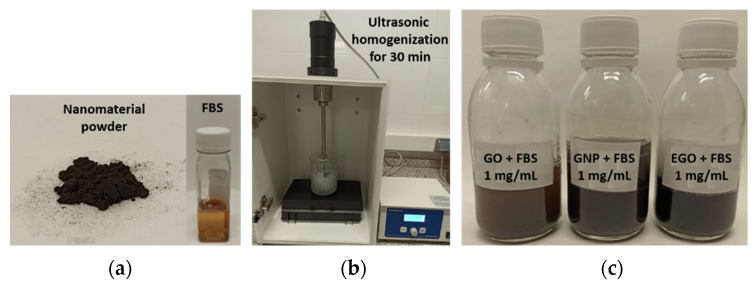
Photographs describing dispersions preparation. (**a**) Nanomaterial powder and FBS; (**b**) homogenization of samples and (**c**) dispersions obtained.

Dispersions of the three graphenoids were prepared (Figure 1) in biological fluid to obtain suspensions with concentrations of 1; 1.5; 2 and 3 mg/mL. Prior to viscosity measurements, the samples were ultrasonicated with an Ultrasonic homogenizer (Bandelin, Germany) equipment for 30 min using 50% of power and a pulsed cycle of 1 s (active and passive intervals of 1 s), in order to obtain a high dispersion and homogenization of the samples. Sonication was carried out by introducing the vessel into a bath with ice water to prevent overheating and degradation of the samples.

### 2.3. Characterization of Samples

In a previous paper, Cerpa et al. performed the characterization of powders GO and EGO using different techniques (IR, Raman, SEM and TEM) [35]. In this work, the structural characterization of nanomaterials and the nanomaterials dispersed in biological fluids were performed with microscopy techniques. Transmission electron microscopy (TEM) images were captured using a JEM-2100 transmission electron microscope (JEM Ltd. Tokyo, Japan) operated at 200 kV in the Centro Nacional de Microscopía (Madrid, Spain). To prepare the TEM samples, dispersions were diluted until 0.01 wt%, and a drop was settled on a copper grid covered with a carbon coating and dried. The samples were also analyzed by scanning electron microscopy (SEM) with a JEOL JSM 6335F, at 20 kV with a secondary electrons detector. One drop of a diluted suspension of each nanoparticle, dispersed either in water or in biologic fluid, was placed on a stainless-steel grid with a graphite layer and dried before analysis. The images of atomic forces microscopy (AFM) of the samples were registered on an AFM multimode Nanoscope III A (Bruker, Germany) of the National Center of Electron Microscopy of the Complutense University of Madrid. A two microliter drop of the dispersion at 3·10^−4^ mg/mL were deposited onto a cleaved muscovite mica substrate and left drying in air for subsequent AFM measurement. Tapping-mode topographic AFM was used.

Zeta potential measurements were performed with a Zetasizer Nano ZS (Malvern, UK) instrument at neutral pH values (6.6 to 7.0), using KCl 0.1 M as inert electrolyte. A pH meter HI5221 (Hanna, Italy) was used to control the pH of the dispersions. The rheological measurements were performed in a Haake RheoStress 6000 rotational rheometer (Thermo Scientific, Karlsruhe, Germany), equipped with a double-cone and plate system (2° cone angle and 60 mm in diameter). A three-stage measuring program was used, with a linear increase in shear rate from 0 to 1000 s^−1^ in 300 s, a plateau at 1000 s^−1^ for 60 s and a further decrease to 0 shear rate in 300 s. Measurements were made for different concentrations of GO, GNP and EGO suspensions (1–3 mg/mL) at temperatures of 25, 30, 37 and 40 ± 0.1 °C, using FBS as dispersing medium and were repeated successively to obtain consistent values. In addition, measurements were made for each nanomaterial dispersed in water and BSA at 1 mg/mL to determine the influence of the dispersion medium on the viscosity values. Due to the limitations of rotational rheometry for low viscosities, the rheological measurements at high shear rates were performed in a rheometer with micrometric capillaries on a chip Fluidicam^Rheo^ (Formulaction, Toulouse, France) at different temperatures in the 25–40 °C range. These measurements are based on the concurrent flow of the sample and a reference liquid of similar viscosity through a Y-shaped tube with a diameter size of 150 μm, thus, allowing the determination of viscosities in the shear rate range from 1000 to near 20,000 s^−1^. 

## 3. Results and Discussion

### 3.1. Transmission and Scanning Electron Microscopy Characterization

**Figure 2:**
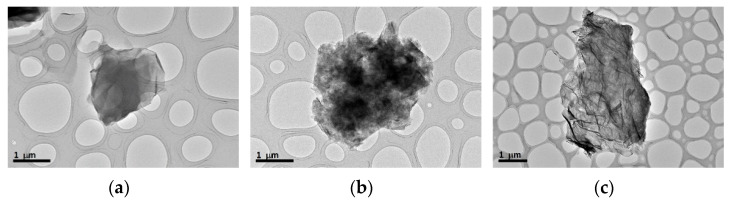
TEM images of the starting carbonaceous materials: (**a**) GO; (**b**) GNP and (**c**) EGO powder.

Structural characterization was carried out using microscopy techniques to determine the shape and size of the nanopowders, as well as the possible morphological changes and interactions between the protein of fetal bovine serum and the carbon nanostructures. Figure 2 shows the images obtained by TEM from the carbon nanostructures used in this work in their as-received powder form. It can be observed that GO displays a thin and transparent sheet, in the case of GNP, the superposition of several layers can be noticed and for EGO, a wrinkled surface with deformed edges due to the formation of folds can be observed. 

**Figure 3:**
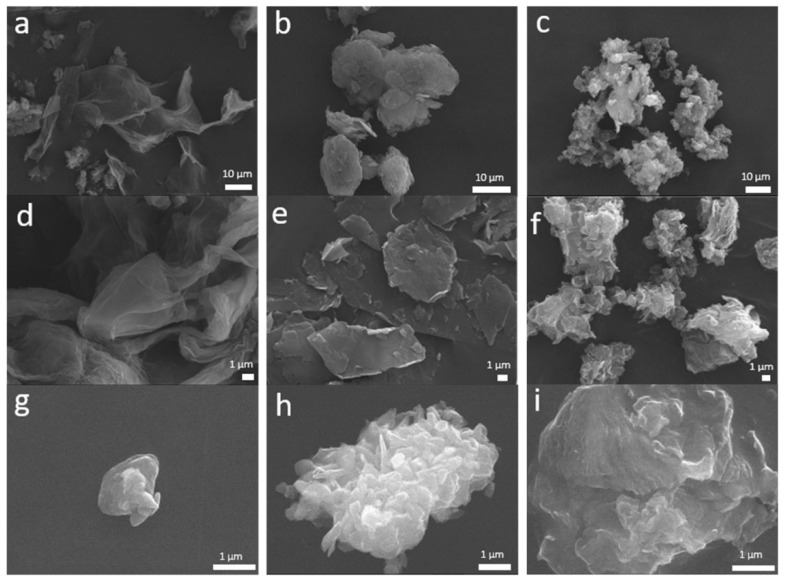
SEM images of GO, GNP and EGO starting powders (**a**,**b**,**c**, respectively) and the same powders dispersed in water (**d**,**e**,**f**, respectively) and dispersed in FBS (**g**,**h**,**i**, respectively).

A more detailed analysis of the particles’ morphology was performed by SEM. Figure 3 shows some marked differences in the morphology of the as received nanopowders, the nanomaterials dispersed in distilled water and those dispersed in fetal bovine serum, as well as the effect of the dispersing media on the structure of those nanomaterials. GO SEM images show a sheet-like structure with a smooth surface, demonstrating that the layers of GO shrink when it is dispersed in water or FBS medium (Figure 3d,g). In the case of GNP and EGO powders, a similar morphology is observed, composed of random aggregates. No significant changes in morphology are observed when these powders are dispersed in water. However, in the case of EGO, it is remarkable that the nanomaterial dispersed in FBS medium forms large agglomerates. Figure 3g–i show a tendency to compaction and aggregation of GO, GNP and EGO when the FBS is used as dispersion medium. This behavior was observed by other authors [36]. Despite this tendency to aggregate, it has been demonstrated that the toxicity of the nanomaterial decreases when it is in contact with the protein solution [37]. 

### 3.2. Atomic Forces Microscopy Characterization

**Figure 4:**
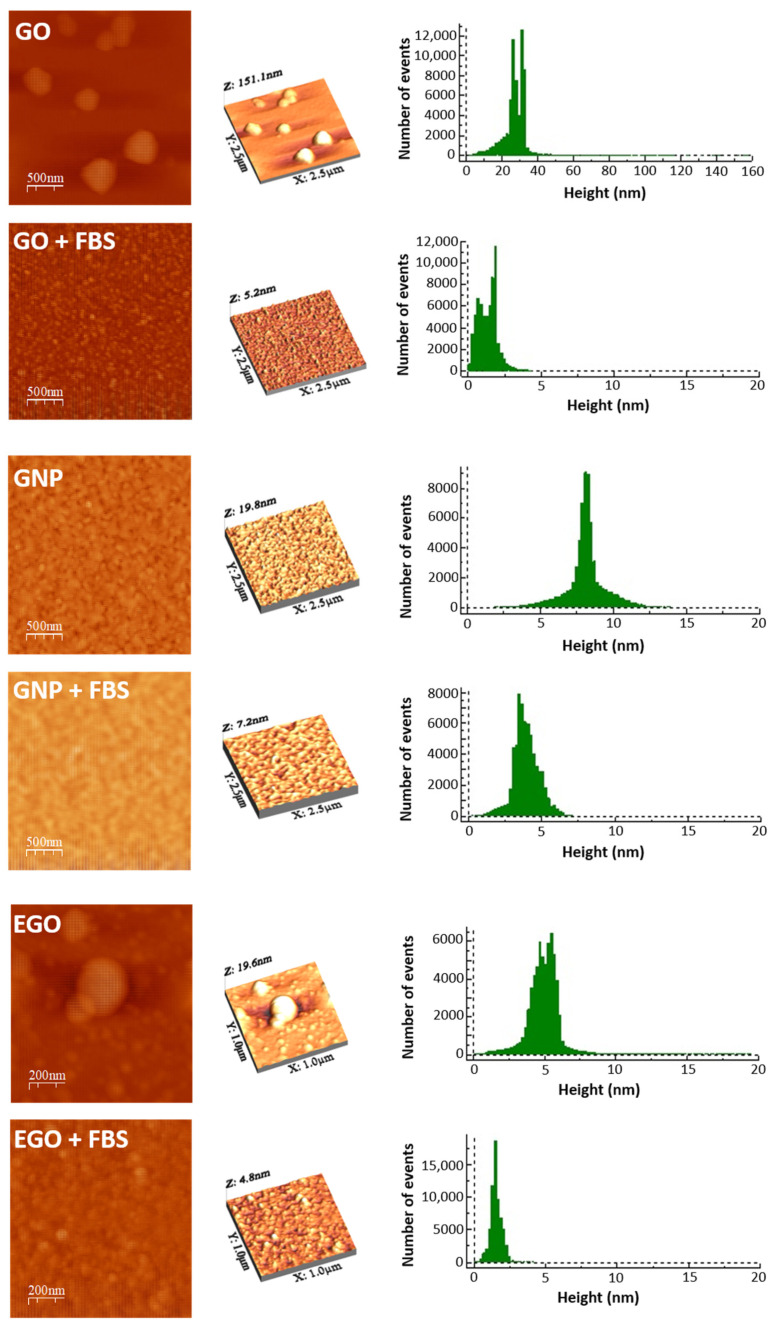
AFM 2D, 3D morphology and high profile of GO, GO + FBS, GNP, GNP + FBS, EGO and EGO + FBS.

The 2D and 3D AFM images of the three graphene-based nanomaterials and their respective morphologies after interacting with the FBS are displayed in Figure 4, where it is also possible to observe the surface texture. These images were processed with the adequate software to obtain the high profile, which is shown at the right hand of the 2D and 3D images, and the root mean square (RMS) roughness that represents the roughness distribution for the entire surface. The average height of GO powder is 27.7 nm, the RMS roughness is 12.7 nm and the mean surface, or average roughness is 5.7 nm, while for GO dispersed in FBS, the textural dimensions experience a drastic decrease, with the average height reduced to 1.4 nm, the RMS roughness to 0.7 nm and the mean surface roughness being only 0.6 nm. The AFM images show how the surface morphology has changed after dispersion in FBS. The AFM images of GNP before and after dispersion in FBS medium indicate that the average height reduces from 8.1 nm for GNP powder to 4.0 nm for GNP + FBS. Regarding the RMS roughness and the mean surface roughness the values for the GNP powders are 1.4 and 0.9 nm, while for the GNP dispersed in FBS these values slightly decrease to 0.9 nm and 0.7. These variations indicate that there is also a good dispersion of the GNP, but lower than that achieved for GO. Finally, for the EGO powder dispersed in FBS, a clear reduction in all the dimensions with respect to the values of the EGO powders is observed. The average height of EGO is 5.1 nm, the RMS roughness is 1.8 nm, and the mean surface roughness is 1 nm. For EGO + FBS, the average height is 1.6 nm, the RMS roughness is 0.4 nm, and the mean surface roughness is 0.3 nm. In summary, it can be stated that for all three nanomaterials, the average height, the RMS roughness, and the mean surface roughness decreased after dispersion in FBS. This demonstrates that the FBS covers the surface of the nanomaterials, decreasing their surface roughness, and therefore suggesting the existence of interaction between protein and nanomaterial [38,39].

### 3.3. Zeta Potential Measurements

Zeta potential measurements allow a quantitative determination of the stability of powders in suspension. Zeta potentials are calculated from the electrophoretic mobility values obtained after three different measurements for each sample. The average of these three measurements with their respective standard deviation are shown in Table 1. The zeta potential value of FBS is −17 mV, which is considered as a reference at pH = 7.4. It is observed that dispersions of GO and EGO have a higher zeta potential absolute value. Different authors accept ±25 mV as an acceptable limit established for assuring the stability of suspensions [40]. Therefore, it is possible to indicate that GO and EGO dispersions are more stable, while GNP dispersions has a higher tendency to aggregation. 

### 3.4. Rheological Studies

The influence of different variables, such as kind of nanomaterial, concentration, temperature, and type of fluid on the rheological properties of the formed dispersions is discussed below.

#### 3.4.1. Type of Graphene-Based Nanomaterial

**Figure 5:**
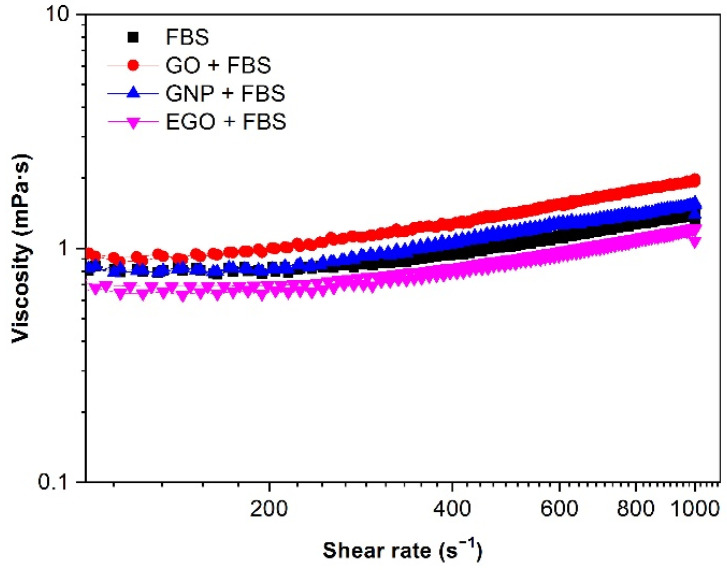
Viscosity (η) vs. shear rate ($\dot{\gamma }$) for 1 mg/mL dispersions of FBS alone and different graphene-based nanomaterials with FBS biological medium. Temperature: 37 °C.

The influence of the different graphene-based nanomaterial on the viscosity in presence of the biologic fluid and fetal bovine serum is shown in Figure 5. The experimental data in all the plots show the mean value of the three data measured for each sample. It is observed that in all cases, for FBS alone and combined with the three nanomaterials, the values of viscosity are extremely low (values are close to water viscosity, 1 mPa·s) and should exhibit a Newtonian flow. However, as it has been discussed in previous reports, rotational rheometry is not adequate for measuring liquids with very low viscosities (less than 3–4 mPa·s) because at high shear rates, there is a strong effect of inertia and wall slippage that results in an apparent but false increase in viscosity in the high shear region [41]. However, rotational rheometry is very well established in many laboratories and is being used elsewhere as a conventional measuring system. The darkness provided by nanographenoid powders complicates the use of other simpler equipments, such as capillary viscometers, in which only transparent liquids can be measured. In addition, these simpler equipments are restricted to purely Newtonian fluids; therefore, they are not indicated for suspensions, where some non-Newtonian behaviour could occur. To obtain a reliable rheological characterization, the samples have been characterized using both a rotational rheometer and a microfluidic chip rheometer. The latter allows the measurement of a sample that is introduced into a capillary together with a reference liquid and the interface can be observed with an optical microscope during the measurement. This method allows one to measure viscosity values up to large shear rate values and complements the measurements obtained with the rotational rheometer.

The FBS curve shows a slightly higher viscosity value than that of EGO + FBS. This may be associated with the use of sonication in the last case, which decreases the intermolecular adhesion forces between EGO and FBS and contributes to particle dispersion. In general, the use of sonication for low content suspensions leads to a better dispersion of the solid phase into the liquid and this is expected to occur in all the studied samples. In fact, this happens for EGO dispersed in FBS and confirms the effect of stabilization observed in the zeta potential values. However, in the case of the dispersion of GO in FBS, the viscosity increases. This could be attributed to a deficient dispersion, but zeta potential measurements demonstrated that the values were the same for GO and EGO, so an external factor should be responsible for this viscosity increase. Since there are no other external parameters, this could be explained by considering that there is an interaction of GO particles with albumin molecules that can promote some agglomeration. Another aspect is that under sonication, the creation of a protein-corona is favoured, as suggested by Raslan et al. [42]. 

#### 3.4.2. Nanomaterial Concentration

**Figure 6:**
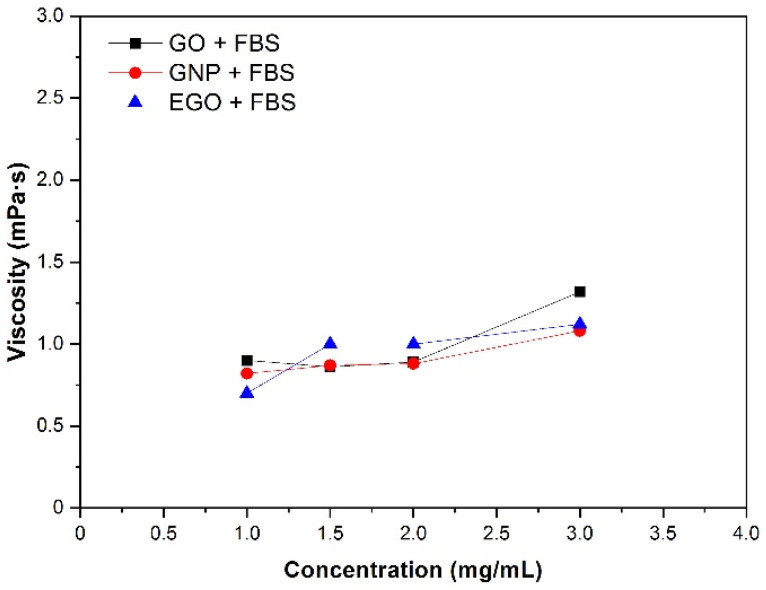
Viscosity of GO, GNP and EGO dispersed in FBS at different nanomaterial concentrations, temperature 37 °C and $\dot{\gamma }$: 200 s^−1^.

The influence of the nanomaterial concentration on the viscosity was studied by keeping the temperature constant at 37 °C and the shear rate at 200 s^−1^. The curves of viscosity obtained for four different concentration dispersions are plotted in Figure 6. The viscosity values obtained are between 0.6 and 1.3 mPa·s for the concentration range of 1–3 mg/mL. These values increase slightly as the concentration increases, although these changes are not significative. The lowest viscosity value is 0.6 mPa·s for the EGO + FBS dispersion at 1 mg/mL, while the highest viscosity value of 1.3 mPa·s corresponds to the GO + FBS dispersion at 3 mg/mL. These values are smaller (0.6–1.3 mPa·s) than obtained by Cerpa et al. for SWCNT and MWCNT (1.8–2.8 mPa·s) [31,32].

#### 3.4.3. Type of Fluid

**Figure 7:**
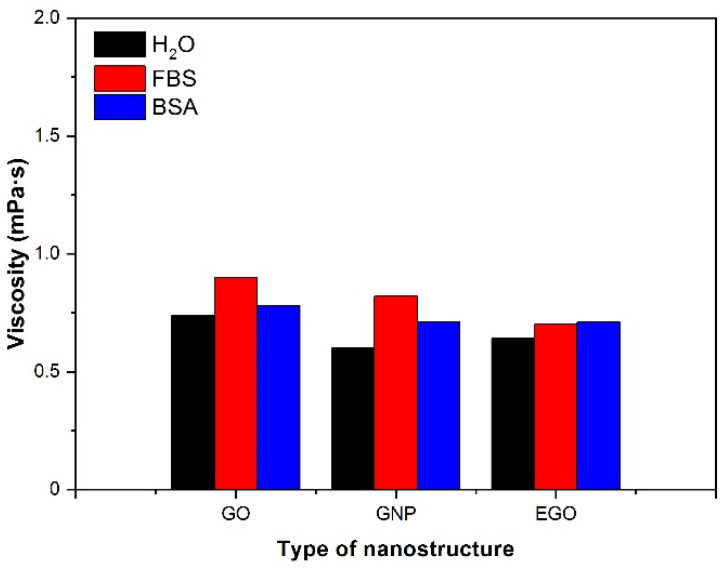
Viscosity values for dispersions of different nanostructures in several fluids. Concentration: 1 mg/mL; temperature 37 °C, $\dot{\gamma }$: 200 s^−1^.

In order to understand the behaviour of the dispersions of nanomaterials in other biological fluids different from FBS, the study was complemented with a few measurements of viscosity in other fluids, such as bovine serum albumin and water. The viscosity of GO, GNP and EGO dispersed in the 3 considered fluids (fetal bovine serum, bovine serum albumin and water) at 1 mg/mL of concentration was measured using the rotational rheometer and values were taken at relatively low shear rates (200 s^−1^) to avoid the apparent shear thickening effect associated with wall slippage. Figure 7 shows the viscosity values with the three fluids for the samples studied at a temperature of 37 °C. When the samples are dispersed in water, the BSA viscosities ranged between 0.6–0.7 mPa·s and 0.7–0.8 mPa·s, respectively. Instead, when FBS is used as dispersion medium, the viscosities range obtained is 0.6–0.9 mPa·s. The dispersion in FBS leads to a slight increase in viscosity in the GO and GNP samples. However, this effect is not observed in the EGO sample because practically the same viscosity value has been obtained for the 3 fluids, approximately 0.7 mPa·s. Similar studies have not been found in the literature for these materials and types of fluids, so there are no reported data for comparison, which justifies the interest of the present investigation. In summary, the viscosity is higher in the presence of FBS for the nanomaterials GO and GNP; however, for EGO, the viscosity presents practically the same value in the presence of the three different fluids.

#### 3.4.4. Effect of Temperature

**Figure 8:**
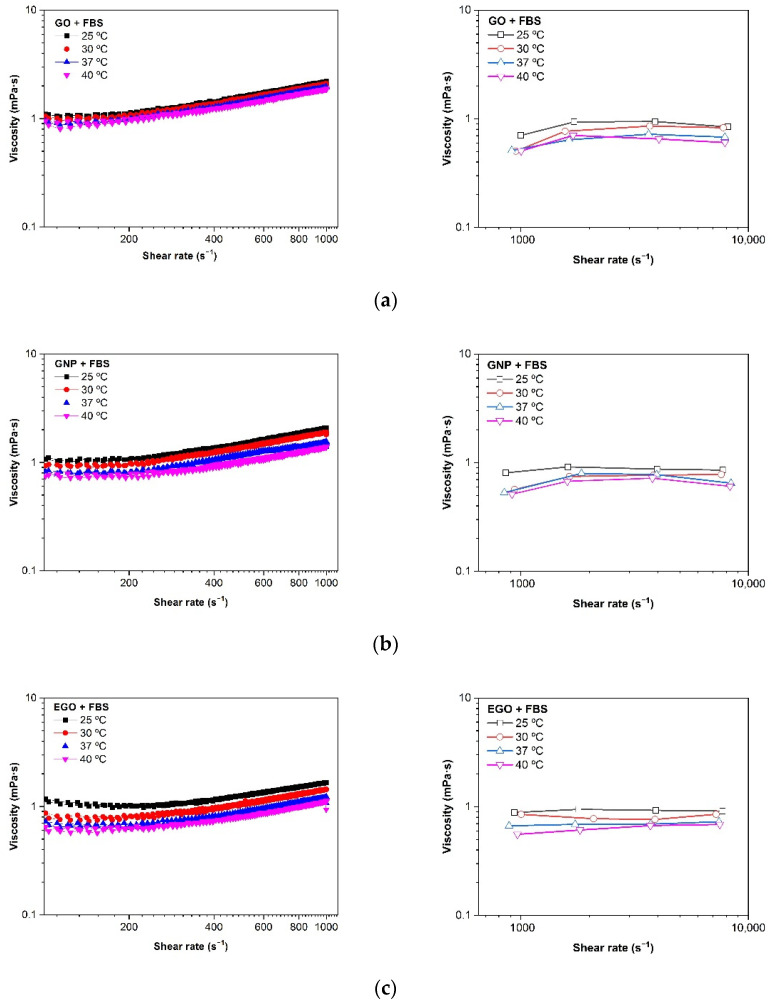
Viscosity (η) vs. shear rate ($\dot{\gamma }$) at different temperatures of (**a**) GO + FBS, (**b**) GNP + FBS and (**c**) EGO + FBS at 1 mg/mL. Rotational rheometer (**left**) and microfluidic chip rheometer (**right**).

The effect of temperature on the viscosity of GO, GNP and EGO dispersed in FBS at a concentration of 1 mg/mL is shown in Table 2 and Figure 8. To determine more precisely the differences in viscosity of the three studied samples, all the measurements were performed using the two rheometers (rotational and microchip fluid). The microfluidic chip rheometer demonstrated to be more precise for low viscous Newtonian fluids; therefore, the measurements could be taken in a wide range of shear rates without deviations from the linearity. It should be noted that, as expected, the viscosity decreases with the increasing temperature because the kinetic energy of the molecules increases, and they become more mobile. Furthermore, the attractive binding energy is reduced, resulting in a lower viscosity [29].

Table 2 shows the viscosity results obtained for each suspension prepared, measured in the rotational rheometer at a shear rate of 200 s^−1^ and measured in the Fluidicam between 1000 and 10,000 s^−1^. The flow curves obtained at a high shear rate show a small increase in the viscosity, as observed in Figure 8. This behaviour has been reported to be an apparent effect associated with the wall slippage during the measurement of very low viscosity liquids, which is the present case with viscosities similar to that of water and is below the error measurement of a rotational rheometer. For this reason, an in-depth analysis of the rheological behaviour was performed using a microfluidic chip rheometer that is mainly used to study fluids with low viscosity at high shear rates [41,43].

The viscosity values measured in the rotational rheometer at low shear rates are slightly higher than those obtained using a microfluidic chip at higher shear rates of 1000–10,000 s^−1^. According to these results, the viscosity values for the GO + FBS, GNP + FBS and EGO + FBS dispersions are between 1.1 and 0.6 mPa·s in the temperature range of 25–40 °C.

However, viscosity values between 1.8 and 1.0 mPa·s for FBS without nanoparticles were obtained at the same temperature range mentioned above. In addition, the viscosity values at temperature of biological interest of 37 °C are between 0.6 and 0.9 mPa·s for the GO + FBS, 0.7–0.8 mPa·s for GNP + FBS and 0.7 mPa·s for the EGO + FBS dispersions, while the viscosity values for FBS at temperature of 37 °C is 1.4–1.1 mPa·s. It can be observed that the type of graphene-based structure does not cause a significant effect on the viscosity when fetal bovine serum is used as the dispersion medium.

Rothammer et al. found that viscosity changed between 1.0 and 1.4 mPa·s within a shear rate range of 10 to 90 s^−1^ [44] for bovine calf serum, with a protein concentration of 20 g/L and a temperature of 37 °C. Contrary to our work, Bortel et al. found a constant viscosity of 0.9 ± 0.05 mPa·s for newborn calf serum of 30 g/L protein concentration within a shear rate range of 1–1000 s^−1^ [45]. Mazzucco et al. also observed Newtonian behavior of bovine serum with 73 mg/mL protein concentration diluted to 40% by volume in distilled water throughout the test range [46], with the viscosity of 1.5 mPa·s.

The measurements obtained with microfluidic technology at higher shear rates (Figure 8, images on the right) clearly confirm the Newtonian behaviour of these dispersions as it was expected, thus, demonstrating that the apparent shear thickening obtained with rotational rheometry is an artefact of the measurement, since the values are within the detection limit of the apparatus and not real behaviour. In addition, there is a very slight difference in the fluid viscosity when the nanomaterial is introduced. So, these nanomaterials (GO, GNP and EGO) at concentrations in the range 1–3 mg/mL do not modify the viscosity of the biological media.

**Figure 9:**
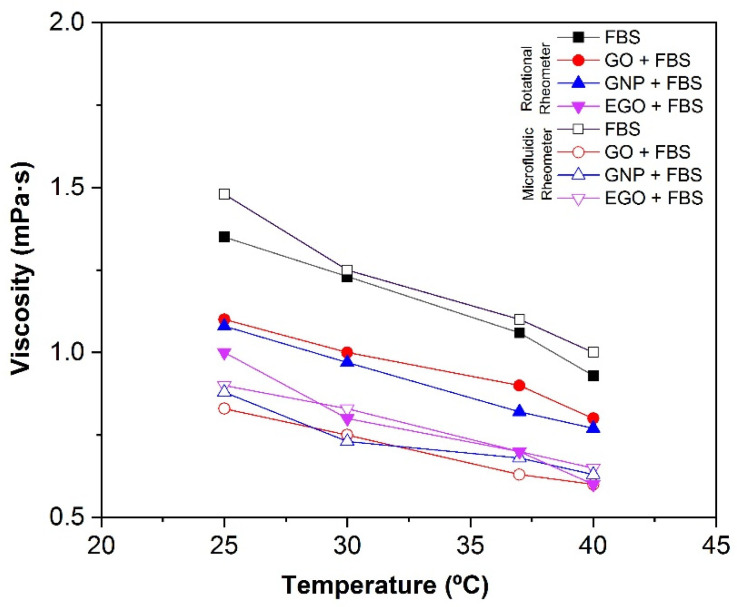
Effect of the temperature on the viscosity of GO + FBS, GNP + FBS and EGO + FBS at 1 mg/mL comparing rotational rheometer and microfluidic rheometer (Fluidicam).

In Figure 9, the effect of temperature in the viscosity measured in both rheometers is shown. The decrease in the viscosity when the temperature increases is proven. It must be remarked the difference in the viscosity obtained from the rotational rheometer and the microfluidic rheometer at 1000 s^−1^. However, the rotational rheometer does not have enough precision for the evaluation of very low viscosities, so the microfluidic chip technique provides a new methodology for the unequivocal determination of such low viscosities. Since the apparent shear thickening occurs at relatively high shear rates where slippage is favoured, in the low shear rate region, the values obtained with both types of rheometers are similar and start to be divergent above 200–300 s^−1^.

## 4. Conclusions

In this work, the rheological behavior of dispersions of three graphenoid nanomaterials in biological fluids has been studied. Variables such as the kind of nanomaterial, its concentration, temperature, and type of fluid have been examined.

- SEM images show a higher tendency to compaction and aggregation of GO, GNP and EGO when the FBS is used as the dispersion medium. The images obtained by AFM show the change in the surface texture, which indicates the nanomaterial–protein interaction.
- The dispersions of GO + FBS, GNP + FBS and EGO + FBS present Newtonian behavior in all the studied ranges of concentration, although measurements performed with rotational rheometers give an apparent shear thickening. The microfluidic chip rheometer is a much more reliable technique for these systems.
- The viscosity values obtained are between 0.6 and 1.3 mPa·s for the concentration range of 1–3 mg/mL at a temperature of 37 °C. These values tend to slightly increase when the concentrations increase, although these changes are not significative.
- The dispersions of nanomaterials in FBS lead to slightly higher viscosity values than those in BSA and water. The viscosity values obtained for GO, GNP and EGO dispersed in FBS at a temperature of 37 °C and a concentration of 1 mg/mL are between 0.6 and 0.9 mPa·s.
- The viscosity of the dispersions decreases with the temperature from 1.1 to 0.6 mPa·s in the temperature range of 25–40 °C at 1 mg/mL.

In summary, this study provides information on the rheological characterization of graphene-based nanomaterials in biological fluids, which can be used in different biomedical applications.

## Figures and Tables

**Table 1:** Zeta potential, mobility, and pH of GO; GNP; EGO suspended in FBS at 1 mg/mL and temperature 25 °C.

Sample	ZP	Mobility	pH
	(mV)	(μm·cm/V·s)	
GO + FBS	−25.7 ± 1.6	−2.0 ± 0.1	6.6
GNP + FBS	−18.1 ± 0.9	−1.4 ± 0.1	7.0
EGO + FBS	−26.6 ± 1.7	−2.1 ± 0.1	6.7

**Table 2:** Viscosity values (mPa·s) of GO; EGO, GNP suspended in FBS at 1 mg/mL in rotational rheometer and Fluidicam equipments.

			Viscosity (mPa·s)			
Sample	Rheometer	Shear Rate (s^−1^)	25 °C	30 °C	37 °C	40 °C
FBS	Fluidicam	1000–10,000	1.5	1.3	1.1	1.0
	Rotational	200	1.8	1.5	1.4	1.3
GO + FBS	Fluidicam	1000–10,000	0.8	0.8	0.6	0.6
	Rotational	200	1.1	1.0	0.9	0.8
GNP + FBS	Fluidicam	1000–10,000	0.9	0.7	0.7	0.6
	Rotational	200	1.0	1.0	0.8	0.8
EGO + FBS	Fluidicam	1000–10,000	0.9	0.8	0.7	0.7
	Rotational	200	1.0	0.8	0.7	0.6

## Data Availability

Not applicable.
